# Genome-Wide Screening of Oxidizing Agent Resistance Genes in *Escherichia coli*

**DOI:** 10.3390/antiox10060861

**Published:** 2021-05-27

**Authors:** Hao Chen, Jessica Wilson, Carson Ercanbrack, Hannah Smith, Qinglei Gan, Chenguang Fan

**Affiliations:** 1 Cell and Molecular Biology Program, University of Arkansas, Fayetteville, AR 72701, USA; hc019@uark.edu (H.C.); jlw098@uark.edu (J.W.); 2 Department of Chemistry and Biochemistry, University of Arkansas, Fayetteville, AR 72701, USA; cwercanb@uark.edu (C.E.); hckriehn@uark.edu (H.S.); qingleig@uark.edu (Q.G.)

**Keywords:** oxidative stress, hydrogen peroxide, hypochlorite, genome-wide screening, AKSA library

## Abstract

The use of oxidizing agents is one of the most favorable approaches to kill bacteria in daily life. However, bacteria have been evolving to survive in the presence of different oxidizing agents. In this study, we aimed to obtain a comprehensive list of genes whose expression can make *Escherichia* *coli* cells resistant to different oxidizing agents. For this purpose, we utilized the ASKA library and performed a genome-wide screening of ~4200 *E. coli* genes. Hydrogen peroxide (H_2_O_2_) and hypochlorite (HOCl) were tested as representative oxidizing agents in this study. To further validate our screening results, we used different *E. coli* strains as host cells to express or inactivate selected resistance genes individually. More than 100 genes obtained in this screening were not known to associate with oxidative stress responses before. Thus, this study is expected to facilitate both basic studies on oxidative stress and the development of antibacterial agents.

## 1. Introduction

In nature, bacteria live under various environmental stresses such as oxidative stress, which is one of the most common challenges for bacteria living in aerobic conditions. Reactive oxygen species (ROSs), reactive nitrogen species (RNSs), and reactive chlorine species (RCSs) are three major sources of oxidative stress [1]. In bacteria, ROSs including superoxide anions (O_2_^•−^), hydrogen peroxide (H_2_O_2_), and hydroxyl radicals (^•^OH) can be formed not only endogenously during electron transfer in the respiratory chain [2], but also exogenously under different conditions such as UV exposure [3]. Although ROSs at certain low concentrations have been demonstrated to be essential for several physiological processes and the cellular redox balance [4], a high level of ROSs has been proven to cause damages to nucleic acids, proteins, fatty acids, and other cellular components [2,3,5]. Different from free radicals such as O_2_^•−^ and ^•^OH, the non-radical ROS H_2_O_2_ is a strong oxidant and has high activation energy, which makes it reactive with transition metal centers, selenoproteins, and selected thiol proteins [6]. An excess presence of H_2_O_2_ has been implicated in the interruption of iron homeostasis in cells [7]. For instance, a high level of H_2_O_2_ can inactivate the *Escherichia coli* Isc iron–sulfur assembly system [8]. Oxidative stress caused by H_2_O_2_ can also induce DNA degradation in bacteria and then inhibit bacterial growth [9]. Differently, hypochlorite (HOCl) mainly targets proteins which contain sulfur, aromatic rings, and primary amines, resulting in protein aggregation and degradation [10]. Due to their disinfecting effects on bacteria, H_2_O_2_ and HOCl, have been commonly used to kill bacteria in daily life. Moreover, antibiotics such as landomycin E, which can induce a rapid generation of H_2_O_2_, have been applied to treat bacterial infections [11,12].

To protect themselves from H_2_O_2_ damages, bacteria have evolved various defense pathways. Besides enzymatic scavengers such as catalase, superoxide dismutase, and peroxidase [13], the stress-induced regulon OxyR is one of the most well-studied approaches used by bacteria to fight against H_2_O_2_ damages [8,14]. As a peroxide sensor and a transcriptional regulator, OxyR can be activated by oxidization at the conserved cysteine residue and trigger the transcription of genes for direct antioxidants such as the *kat* family (catalases), *sodA* (superoxide dismutase), and *ahpCF* (alkyl hydroperoxide reductase), as well as other proteins involved in amino acid biosynthesis and cell wall synthesis [15,16]. For HOCl, bacteria have developed different defense mechanisms, such as converting Hsp33, RidA, and CnoX into highly active chaperones to prevent proteins from aggregation and degradation caused by HOCl [17,18]. HOCl-responsive transcriptional factors such as HypT, RclR, and NemR can also help to alleviate HOCl damages [19]. For instance, HypT can be activated by oxidation of its methionine residues and then upregulate the transcription of genes participating in the biosynthesis of amino acids such as methionine (*metB*, *metK*, and *metN*), while downregulating intracellular iron levels [20]. Interestingly, recent studies have demonstrated that noncoding RNAs and small proteins are also involved in oxidative stress responses, which has never been recognized before [13]. Therefore, it is necessary to explore unknown mechanisms of oxidative stress responses in bacteria to provide new information for antibacterial agent development.

Previous studies on oxidative stress responses have mostly focused on analyzing gene expression and regulation in cells after exposure to oxidizing agents. Some genes associated with oxidative stress responses might not be expressed under experimental conditions. Thus, we aimed to obtain a comprehensive list of genes whose expression can make cells resistant to oxidizing agents. In this study, we used *E. coli* as the representative bacterium, while H_2_O_2_ and HOCl were selected as oxidizing agents. The ASKA collection, which is a complete set of *E. coli* strains for overexpressing individual *E. coli* K-12 genes, was screened to identify oxidizing agent resistance genes. Different from most screening studies with the ASKA library which used either pooled strains or plasmids [21,22], we tested all the strains individually to avoid potential interferences by pooling them together. Additionally, we further validated candidate genes from the screening in the common *E. coli* K-12 MG1655 strain to avoid potential impacts of the host strain of the ASKA library, the AG1 strain, which has been engineered for high transformation efficiency. In our list of genes, there are several genes which were known for their functions or association with oxidative-stress responses. Further studies are ongoing to explore their specific mechanisms.

## 2. Materials and Methods

### 2.1. Chemicals and Bacterial Strains

All the chemicals, reagents, bacterial growth media such as H_2_O_2_ and NaOCl solution, LB medium, M9 medium, chloramphenicol, β-D-1-thiogalactopyranoside (IPTG) were purchased from VWR International (Radnor, PA, USA). The ASKA library and Keio collection were originally from the Coli Genetic Stock Center at Yale University. The no-insert control of pCA24N was from our previous study [23]. The plasmids with candidate genes were purified from candidate strains individually by Qiagen Miniprep plasmid purification kits (Hilden, Germany) and transformed into MG1655 cells by Bio-Rad Pulser™ Transformation Apparatus (Hercules, CA, USA).

### 2.2. ASKA Library Screening and Minimal Inhibitory Concentration (MIC) Determination

Individual plates of the ASKA library were replicated by inoculating 5 µL stock culture into 200 µL fresh LB medium with 50 µg/mL chloramphenicol in each well of the new plates and incubating them at 37 °C overnight. The absorbance at 600 nm of each well was read by a microplate reader. On the next day, the overnight culture in each well was diluted to OD600 nm = 0.15 with a total volume of 200 µL fresh LB medium, 50 µg/mL chloramphenicol, 0.1 mM IPTG, and corresponding concentrations of H_2_O_2_ or NaOCl. Each plate had three biological replicates. The 96-well plates were sealed with oxygen-permeable membranes (Sigma-Aldrich, St. Louis, MO, USA). Cell growth was monitored by reading the absorbance at 600 nm with microplate readers at 37 °C, continuously. MIC determination was performed by using varying concentrations of oxidizing agents with 1 mM increments from 1 to 20 mM, or 0.25 mM increments from 0 to 1 mM in LB or M9 minimal medium. The lowest concentration at which bacteria could not grow was recorded as the corresponding MICs.

### 2.3. Bioinformatical Analyses

The subcellular localization of proteins was obtained from the EcoCyc *E. coli* Database by typing individual gene names into the database [24]. The identified proteins were classified into functional categories according to their annotated functions in the UniProt-GOA Database [25] and analyzed by DAVID Bioinformatics Resources by typing individual gene names into the database [26]. Protein–protein functional interaction networks were analyzed with the STRING database [27], in which active interaction sources from experiments, databases, co-expression, neighborhood, gene fusion, and co-occurrence were selected. The minimum required interaction score of medium confidence was chosen.

## 3. Results

### 3.1. Genome-Wide Screening of Resistance Genes against H_2_O_2_

First, we determined the MIC of H_2_O_2_ for the no-insert control strain of the ASKA library (the AG1 strain with pCA24N empty vector). MIC was 2 mM. Then, we used a 2-fold MIC (4 mM H_2_O_2_) to screen the complete ASKA library for H_2_O_2_ resistance genes. Each strain was tested three times. Only those growing in all three replicates were recorded as candidates for further analyses. There were 217 candidate genes after the screening. We listed them into categories based on their biological functions (Table 1).

**Figure 1:**
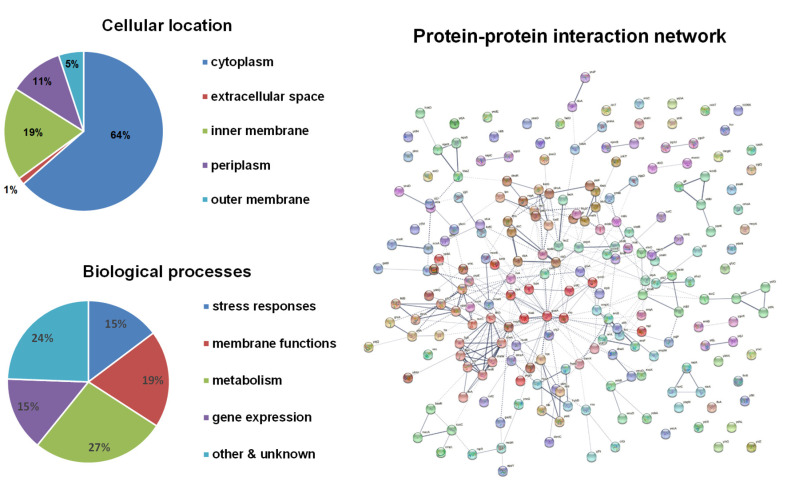
Bioinformatical analyses of H_2_O_2_-resistance genes. The subcellular localization of proteins was obtained from the EcoCyc *E. coli* Database. The identified proteins were classified into functional categories according to their annotated functions in the UniProt-GOA Database and analyzed by DAVID Bioinformatics Resources. Protein–protein functional interaction networks were analyzed with the STRING database. A high-resolution interaction map is shown in Figure S1.

Bioinformatical analyses of these candidate genes were performed (Figure 1). About two-thirds of the gene products are in the cytoplasm, leaving the other one-third associated with membranes. The functions of these genes cover a wide range of biological processes and are enriched in four major categories: stress responses, membrane functions (mostly transporters), metabolism, and gene expression and regulation. The protein–protein interaction network showed four major clusters: (1) metabolic enzymes including *pflD* (formate acetyltransferase), *ttdB* (tartrate dehydratase), *dmlA* (malate dehydrogenase), *ybiW* (formate acetyltransferase), *sucC* (succinyl-CoA ligase), *sucA* (2-oxoglutarate dehydrogenase), *leuA* (2-isopropylmalate synthase), and *prpE* (propionyl-CoA ligase); (2) flagellar biosynthesis and mobility including *flhD*, *flgE*, *flgK*, *fliS*, *motB*, *cheW*, and *cheA*; (3) protein synthesis including *thrA* (threonine synthesis), *hisB* (histidine synthesis), *ilvA* (threonine synthesis), *guaA* (GMP synthase), *rho* (transcription termination), *yidC* (ATP synthase), and *rpmG* (50S ribosomal protein L33); (4) oxidative stress responses including *sodB* (superoxide dismutase), *katE* (catalase), *btuE* (Thioredoxin/glutathione peroxidase), *katG* (catalase-peroxidase), *tpx* (thiol peroxidase), and *galU* (UTP-glucose-1-phosphate uridylyltransferase).

To identify genes which mediate stronger resistance to H_2_O_2_, we used a 3-fold MIC for the wild-type strain (6 mM H_2_O_2_) to screen those 217 candidate genes. Only 20 strains could grow, including *appY*, *citG*, *damX*, *ilvA*, *katE*, *katG*, *kefC*, *leuA*, *metN*, *prpE*, *rho*, *sapC*, *yajQ*, *ybhC*, *ydhL*, *yhjJ*, *yncC*, *yncG*, *yqhA*, and *yrbB*, which were selected for further validation. A summary of the current knowledge about these genes is shown in Table 2. Besides *katE*, *katG*, and *yncG*, which are oxidative stress response genes, most genes play roles in either membrane transport or gene expression and regulation. More details are described in the Discussion section.

### 3.2. Genome-Wide Screening of Resistance Genes against HOCl

Similar screenings were performed with HOCl (in the form of NaOCl) as the oxidizing agent. The MIC was 1 mM for the no-insert control strain. Then, we used a 2-fold MIC (2 mM NaOCl) to screen the whole ASKA library. Compared to H_2_O_2_, there were fewer resistance genes for HOCl. Only 114 strains could grow in 2 mM NaOCl. We divided them into categories based on their biological functions (Table 3).

**Figure 2:**
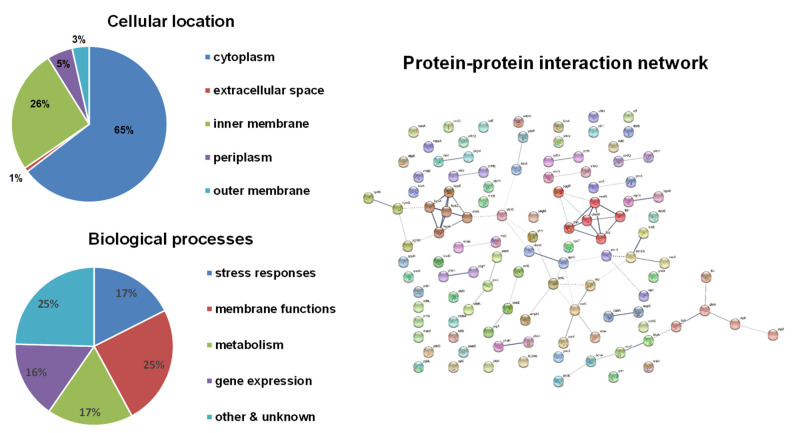
Bioinformatical analyses of HOCl-resistance genes. The subcellular localization of proteins was obtained from the EcoCyc E. coli Database. The identified proteins were classified into functional categories according to their annotated functions in the UniProt-GOA Database and analyzed by DAVID Bioinformatics Resources. Protein–protein functional interaction networks were analyzed with the STRING database. A high-resolution interaction map is shown in Figure S2.

Bioinformatical analyses were performed on these candidate genes (Figure 2). Compared to the H_2_O_2_ results, a higher number of inner membrane-associated proteins appeared to be coded by HOCl-resistance genes. Similar to H_2_O_2_-resistance genes, the functions of these candidate genes also cover a wide range of biological processes and focus on stress responses, membrane functions, metabolism, and gene expression. A little higher proportion of genes involved in membrane functions were identified for HOCl-resistance. The protein–protein interaction network only showed two clusters: (1) the respiration chain including hydrogenase (*hyaA*, *hyaC*, *hyaE*, *hyaF*) and ubiquinol oxidase (*cbdA*); (2) flagellar biosynthesis and mobility including *fliP*, *fliS*, *motB*, *cheW*, and *tap*, which was found also in H_2_O_2_-resistance genes.

To identify genes which mediate stronger resistance to HOCl, we used a 3-fold MIC for the wide-type strain (3 mM NaOCl) to screen the identified 114 candidate genes. Only 23 strains could grow, which expressed *elaA*, *exbD*, *frmB*, *hyaC*, *hyaE*, *ilvA*, *marC*, *motB*, *prpE*, *rnc*, *rsuA*, *sanA*, *sucC*, *tap*, *ybhR*, *ycbB*, *yccJ*, *ycdK*, *yedQ*, *yfdY*, *yhdJ*, *yoaE*, and *ytfI*. They were selected for further validation. A summary of the current knowledge about these genes is presented in Table 4. Similar to the H_2_O_2_ results, membrane transport and gene expression are also two important functions of HOCl-resistance genes. Furthermore, cellular redox balances and DNA damage responses are two unique processes of HOCl-resistance genes. More details are described in the Discussion section.

### 3.3. Effects of Overexpressing Selected Resistance Genes in the MG1655 Strain

**Figure 3:**
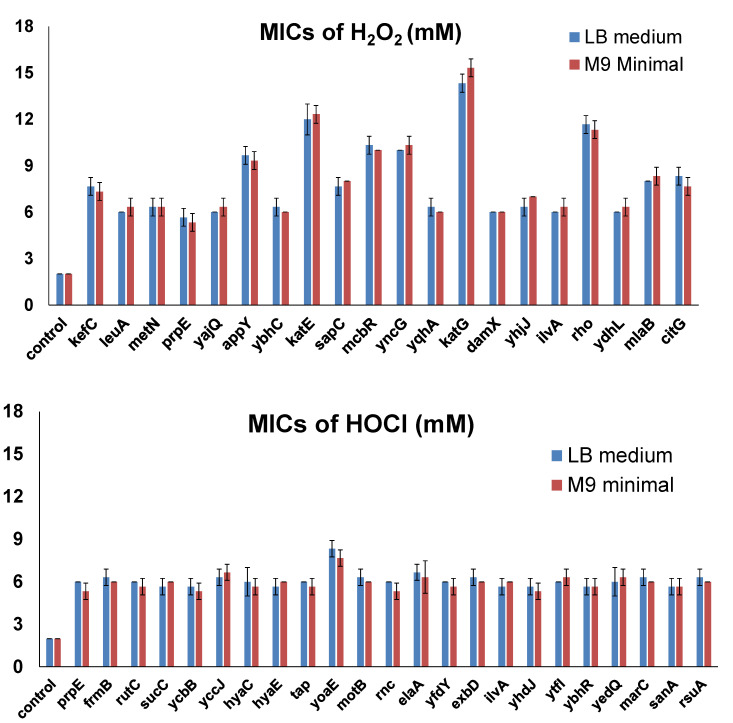
MICs of selected genes overexpressed in MG1655. MIC determination was performed by varying the concentration of the oxidizing agents, with 1 mM increments from 1 to 20 mM in the LB medium or the M9 minimal medium. The lowest concentration at which bacteria could not grow was recorded as the corresponding MIC. Each strain was tested in three biological replicates. All the differences between MICs of the candidate genes and those of the control were highly significant (*p* < 0.001).

The host strain for the ASKA library is AG1, which was engineered for high transformation efficiency. Therefore, the AG1 strain may have potential inferences for the screening. To validate the effects of selected candidate genes which made cells resistant to concentrations that were 3-fold the MICs for wild-type cells in the first screening, we overexpressed each of them (20 genes for H_2_O_2_ reported in Table 2 and 23 genes for HOCl reported in Table 4) in the K-12 strain MG1655, which is commonly used for *E. coli* physiology studies. The LB medium is a rich medium containing amino acids that react with oxidizing agents to potentially affect cellular responses. Therefore, we used both the LB medium and the M9 minimal medium (0.2% glucose) to determine MICs in the validation experiments. MICs of H_2_O_2_ and NaOCl for the no-insert control strain (MG1655 with pCA24N empty vector) were 2 mM for H_2_O_2_ and 2 mM for NaOCl. Then, we determined the MICs of H_2_O_2_ or NaOCl for each resistance gene overexpressed in MG1655 cells (Figure 3). All the genes did not show significant differences in the MICs using the LB medium and the M9 minimal medium. The results were consistent with those of the ASKA library screening. All candidate resistance genes allowed MG1655 cells to grow at concentrations at least 3-fold the MICs for the control strain. For H_2_O_2_, there were 10 genes that allowed cells to grow in the presence of even higher concentrations. Besides *katE* and *katG*, which encode two known catalases, overexpression of *rho* made cells resistant to 12 mM H_2_O_2_, while overexpression of *appY*, *mcbR*, or *yncG* increased the MIC to 5-fold (10 mM H_2_O_2_) of the MIC for the control strain. On the other hand, overexpression of only one gene (*yoaE*) required a slightly higher MIC (8 mM NaOCl) for HOCl resistance.

### 3.4. Effects of Inactivating Selected Resistance Genes in the E. coli Genome

**Figure 4:**
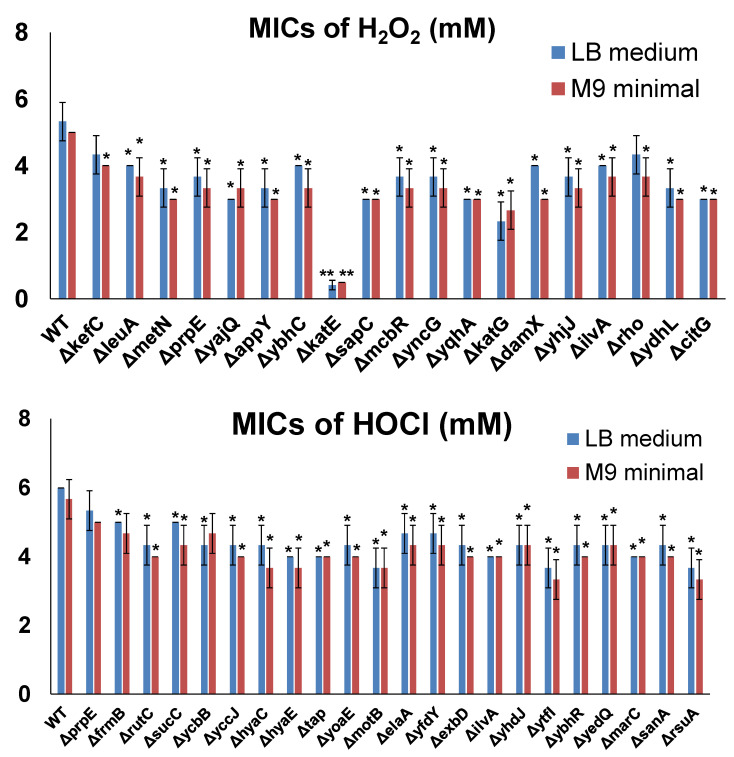
MICs of selected genes inactivated in BW25113 cells from the Keio collection. MIC determination was performed by varying the concentration of the oxidizing agents, with 1 mM increments from 1 to 10 mM for both H_2_O_2_ and HOCl and 0.25 mM increments from 0 to 1 mM (if MICs were lower than 1 mM) in LB medium or M9 minimal medium. The lowest concentration at which bacteria could not grow was recorded as the corresponding MIC. Each strain was tested in three biological replicates. Significant differences (*p* < 0.05) are marked with *, and highly significant differences (*p* < 0.001) are marked with **.

To further confirm the resistance induced by the selected genes in MG1655 overexpression tests, we determined the MICs of H_2_O_2_ or NaOCl in *E. coli* cells by inactivating each individual gene. For this purpose, we utilized the Keio collection, which contains strains with each non-essential *E. coli* gene inactivated. Firstly, we determined the MICs of H_2_O_2_ or NaOCl for the wild-type control of the Keio collection, i.e., the BW25113 strain. The MICs of BW25113 cells were 5 mM for H_2_O_2_ and 6 mM for NaOCl. Then, we determined the MICs of H_2_O_2_ or NaOCl for each resistance gene inactivated in BW25113 cells (Figure 4). As shown, inactivation of most candidate genes (except for *prpE* for HOCl resistance) made *E. coli* cells more sensitive to H_2_O_2_ or NaOCl compared to the wild-type strain, confirming that resistance genes identified from the ASKA library could help cells survive in the presence of oxidizing agents. Compared to gene overexpression testing, in which all the candidates showed highly significant effects (Figure 3), gene inactivation testing did not produce highly significant effects (except for *∆katE*). This observation indicates that there are other defense mechanisms which can compensate for the inactivated resistance genes.

## 4. Discussion

### 4.1. Summary of the Study

Aiming to identify genes in the whole genome of *E. coli* cells whose expression can induce resistance to H_2_O_2_ or HOCl, this study utilized the ASKA library and further validated candidate genes in common *E. coli* K-12 strains. In total, ~4200 ORFs from the ASKA library were tested individually. Besides some well-known genes such as *katG* and *katE* for oxidative stress responses, this study identified a number of genes (105 genes for H_2_O_2_ and 63 genes for HOCl) which had not been shown to associate with oxidative stress responses before. On the other hand, some well-known response genes such as *oxyR* for H_2_O_2_ responses and *hypT* for HOCl responses were not identified in our study. To confirm this result, we determined the MICs of MG1655 cells expressing *oxyR* or *hypT* individually using the same protocol as that for candidate genes. The results showed that the engineered cells had the same MICs as those of WT MG1655 cells. One possible reason is that these response proteins are activated upon oxidation by oxidizing agents [20,62] and, when they were overexpressed in our screening, the average oxidation stoichiometry decreased below the level necessary for their activation. Thus, our study nicely complements previous oxidative stress studies, providing new information in this field.

We identified 217 candidate genes for H_2_O_2_ resistance and 114 candidate genes for HOCl resistance from our genome-wide screening. Only 27 genes were identified in both sets of candidate genes, including *agar*, *agaS*, *cheW*, *exbD*, *fliS*, *gloA*, *hyaA*, *ilvA*, *leuA*, *motB*, *nrfC*, *prpE*, *sbmC*, *slyB*, *sucC*, *ubiD*, *upp*, *yagM*, *ybhC*, *ydeQ*, *ydfB*, *ydfD*, *ydhL*, *yhaJ*, *yodA*, *yoeE*, and *yrbB*. A summary of the current knowledge of these genes is presented in Table 5. Most of them are involved in stress responses, gene expression, membrane transport, and cell mobility. These overlaps indicate shared mechanisms in oxidative stress responses. However, we also found a large number of genes specific for H_2_O_2_ or HOCl, indicating distinct mechanisms in oxidative stress responses. Further studies will be implemented to explore the mechanisms of the genes associated with oxidative stress responses.

### 4.2. H_2_O_2_-Resistance Genes

Twenty genes were identified to mediate stronger H_2_O_2_ resistance (Table 2). Among them, 13 genes encode proteins which function in the cytosol, while the others encode proteins located in cell membranes. Most of these genes have been functionally studied. Besides two well-known catalase genes *katG* and *katE*, the functions of other genes are diverse. For instance, *appY* and *mcbR* function as transcriptional regulators; *leuA*, *prpE*, *ilvA*, and *citG* encode enzymes involved in cell metabolism; *kefC*, *metN*, *sapC*, *yqhA*, and *mlaB* encode membrane proteins.

Unsurprisingly, *katG* and *katE* in *E. coli* induce stronger H_2_O_2_ resistance than other genes. Interestingly, although the strain with *katG* overexpression showed a higher MIC (15 mM) than the one overexpressing *katE* (12 mM), the *ΔkatE* strain (MIC 0.5 mM) apeared more sensitive to H_2_O_2_ than the *ΔkatG* strain (MIC 2 mM). One possible explanation for this paradox is that a threshold concentration of H_2_O_2_ is required for *katG* expression [76]. Moreover, the *katG* gene is regulated by the OxyR regulon [77], while the expression of the *katE* gene is permanently induced in aerobic environment [78]. Thus, *katE* can quickly protect cells when *katG* is inactivated.

The gene *rho* encodes the transcription termination factor Rho, which is responsible for the termination of over half of the transcripts [79] and is related to several important physiological processes in *E. coli* [42]. It mediates a strong H_2_O_2_ resistance (MIC 12 mM) when overexpressed but is not very sensitive to H_2_O_2_ when inactivated. This could be explained by the previous finding that the activity of Rho in bacteria could be altered under stressful conditions [80]. Overexpression of *rho* could compensate the negative effects brought by a dysfunctional Rho under stress conditions.

The transcriptional regulator OxyR has been known as the major regulon for responses to H_2_O_2_ stress [14]. In this study, two more transcriptional regulators, AppY and McbR, were also identified. The overexpression of *appY* and *mcbR* allowed *E. coli* cells to grow in 10 mM H_2_O_2_. AppY was found to function as a transcriptional activator of energy metabolism genes under stressful conditions such as anaerobiosis and phosphate starvation [33]. It was reported that a AppY-defective *E. coli* strain was more sensitive to H_2_O_2_ than the wild-type strain [81], which is consistent with our result. On the other hand, McbR has been demonstrated to be involved in H_2_O_2_ responses in avian pathogenic *E. coli* by downregulating the expression of the stress response genes *yciF* and *yciE* [82].

In addition to the genes discussed above, *yncG*, a gene for a putative glutathione S-transferase, was also shown to induce obvious H_2_O_2_ resistance in our tests. Although one previous study demonstrated that YncG does not exhibit GSH activity when expressed in cell-free systems [38], YncG may have a different function in vivo, i.e., a GSH-dependent peroxidase activity similar to that of another putative glutathione S-transferase, GST B1-1 [83].

### 4.3. HOCl-Resistance Genes

Twenty-three genes were identified to mediate stronger HOCl resistance (Table 4). Among these genes, 12 genes encode proteins functioning in the cytosol, while the others encode proteins located in membranes. Surprisingly, all of these 23 genes have not been mentioned as parts of any known HOCl response mechanisms. Some of them have been indicated to be activated under stress conditions. For example, *ycbB* encodes the L,D-transpeptidase with a role in protecting outer membranes during cell envelope stress [48]; The *yedQ* gene encodes a probable inner membrane protein with predicted diguanylate cyclase activity [59]. Expression of *sanA* is implicated in strengthening membrane permeability in stationary-phase stress responses [60]. It is reasonable that these three genes encode proteins in membrane systems, as HOCl has been found to damage the cell envelope system of bacteria [19]. Besides them, we also identified eight genes (*marC*, *ybhR*, *exbD*, *yfdY*, *motB*, *yoaE*, *hyaC*, and *tap*), which encode proteins in membrane systems. Among them, *yfdY* was indicated as a participant in biofilm formation, which is a defense mechanism against HOCl in *E. coli* [55].

In addition to genes coding for membrane-associated proteins, we also identified genes involved in other biological processes such as metabolism (*sucC* in the citric acid cycle; *prpE* in propionate metabolism) [31], amino acid synthesis (*ilvA* in isoleucine biosynthesis) [41], DNA and RNA modifications (*yhdJ* for methylation of genomic DNA; *rsuA* for pseudouridylation of 16S rRNA) [57,61], and rRNA processing (*rnc*) [54]. One possible mechanism is that overexpression of these proteins could compensate for their corresponding native proteins which are inactivated by oxidation.

Different from the results for H_2_O_2_, MIC determination tests showed no significant differences among the 23 candidate genes for HOCl. Only *yoaE*, which encodes a putative transport protein, induced slightly stronger HOCl resistance than other genes. Although there is no previous report indicating the role of YoaE in *E. coli* stress responses, a recent study demonstrated that the expression of the *yoaE* gene in *Salmonella enterica* could be upregulated by CpxR, which plays an important role in repairing bacterial envelope damages [84].

## 5. Conclusions

In this study, we performed genome-wide screening of the *E. coli* ASKA collection and identified 217 candidate genes for H_2_O_2_ resistance and 114 candidate genes for HOCl resistance. Among them, 105 genes for H_2_O_2_ and 63 genes for HOCl were not shown to associate with oxidative stress responses before. Further studies are necessary to validate the genes here identified, which appear as promising new candidates for oxidative stress studies. Furthermore, because the disinfecting mechanisms of many antibiotics are related to oxidative stress, this study is expected to facilitate antibiotic development.

## Figures and Tables

**Table 1:** List of H_2_O_2_-resistance genes from the genome-wide screening.

Biological Functions ^1^	Genes ^2^
Stress responses	*btuE*, *cheA*, *cheW*, *evgS*, *exoX*, *katE*, *katG*, *mepA*, *osmE*, *phoA*, *psiF*, *radA*, *rarA*, *ruvC*, *sbmC*, *sodB*, *sufC*, *tpx*, *ttdB*, *yajQ*, *ybcM*, *ybhT (acrZ)*, *ybiJ*, *ycdO (efeO)*, *ycjU (pgmB)*, *ydjR (ves)*, *yeaD*, *yegD*, *yfiD (grcA)*, *yhiO (uspB)*, *yjjX*, *yodA (zinT)*, *ypdA (pyrS)*, *yraJ*
Membrane components and transporters	*amiB*, *btuD*, *cmtB*, *cusC*, *damX*, *dmsA*, *exbD*, *feoB*, *fhuA*, *frvB*, *ftsH*, *gatC*, *hyaA*, *kefB*, *kefC*, *macA*, *metN*, *mltC*, *mtlA*, *nrfC*, *nuoB*, *ompL*, *ompW*, *ompX*, *oppD*, *rbsA*, *rfbX*, *sapC*, *shiA*, *slyB*, *wcaD*, *ybhC*, *ycjP*, *ydcT*, *ygjQ*, *yidC*, *yobA*, *yrbB (mlaB)*
Metabolism	*aceA*, *aceK*, *agaS*, *allB*, *aroB*, *aroF*, *aroL*, *astD*, *citG*, *cobT*, *eda*, *fadD*, *galU*, *gatD*, *gloA*, *guaA*, *hcr*, *hisB*, *hpt*, *hybD*, *hypB*, *ilvA*, *ispA*, *kbaZ*, *kdsA*, *lacA*, *lacZ*, *leuA*, *lpcA (gmhA)*, *moaA*, *moeB*, *paaB*, *pflA*, *pflD*, *phnI*, *prpD*, *prpE*, *puuD*, *ribA*, *ribC*, *ribD*, *sucA*, *sucC*, *tdk*, *thiL*, *thrA*, *ubiD*, *upp*, *wbbI*, *yaiE (ppnP)*, *ybdK*, *ybiS (ldtB)*, *ycfS (idtC)*, *ydeN*, *ydiR*, *ydjA*, *yeaU (dmlA)*, *yfbB (menH)*
DNA replication, gene expression and regulation	*agaR*, *appY*, *baeR*, *cpdA*, *deoR*, *dnaX*, *flhD*, *fnr*, *gadE*, *mngR*, *pepA*, *rho*, *rluA*, *rna*, *rpmG*, *rsgA*, *stpA*, *tus*, *umuD*, *valS*, *ydfH*, *yecO (cmoA)*, *yggD (fumE)*, *yhaJ*, *yidZ*, *yjeK (epmB)*, *yjfN*, *yncC (mcbR)*, *yraO (diaA)*
Cell division and mobility	*flgE*, *flgK*, *fliS*, *flxA*, *minD*, *minE*, *motB*, *ybgF*, *ydeQ*, *yfiR*, *yihG*, *yraP*
Other and unknown	*glf*, *hokD*, *mpl*, *yabQ*, *yagM*, *yaiX*, *ybcH*, *ybfH*, *ybhN*, *ybiI*, *ybiV*, *ybiW*, *yceH*, *ycgL*, *yciC*, *yciK*, *yddK*, *ydfA*, *ydfB*, *ydfD*, *ydhL*, *ydjJ*, *ydjL*, *yeaO*, *yecA*, *yecI*, *yedE*, *yedM*, *yfdM*, *yfjM*, *ygcP*, *ygcU*, *ygeR*, *yhjJ*, *yihM*, *yjcF*, *yjjJ*, *ykfH*, *ymcB*, *yncG*, *yoaH*, *yobB*, *yoeE*, *yqeK*, *yqhA*, *yrbL*

^1^ Genes were classified into functional categories according to their annotated functions in the UniProt-GOA Database [25]. ^2^ Gene Synonyms are listed in parentheses.

**Table 2:** List of genes mediating stronger H_2_O_2_ resistance.

Gene	Known or Projected Functions
*kefC*	K+: H+ antiporter; plays a role in protecting the cell from electrophile toxicity [28].
*leuA*	2-isopropylmalate synthase; involved in the first step of leucine biosynthesis [29].
*metN*	L-methionine/D-methionine ABC transporter ATP-binding subunit [30].
*prpE*	Propionyl-CoA synthetase; catalyzes formation of propionyl-CoA, the first reaction in propionate catabolism via the methylcitrate cycle [31].
*yajQ*	A nucleotide binding protein [32].
*appY*	DNA-binding transcriptional activator; induces the expression of energy metabolism genes under anaerobiosis, stationary phase, and phosphate starvation [33].
*ybhC*	An outer membrane lipoprotein [34].
*katE*	Catalase HPII; the primary scavenger at high H_2_O_2_ concentrations [35].
*sapC*	Putrescine ABC exporter membrane protein; putrescine efflux [36].
*mcbR*	A member of the FadR C-terminal domain (FCD) family in the GntR superfamily of transcriptional regulators [37].
*yncG*	Putative glutathione S-transferase [38].
*yqhA*	Uncharacterized protein; predicted to be an integral membrane protein.
*katG*	Catalase/hydroperoxidase; bifunctional with both catalase and peroxidase activity [39].
*damX*	Non-essential cell division protein [40].
*yhjJ*	Peptidase M16 family protein.
*ilvA*	Threonine deaminase; carries out the first step in the synthesis of isoleucine [41].
*rho*	Transcription termination factor; required for one of the two major types of termination of RNA transcription [42].
*ydhL*	DUF1289 domain-containing protein.
*mlaB*	Intermembrane phospholipid transport system protein; forms a stable complex with MlaF, MlaE, and MlaD and is required for the stability of this complex [43].
*citG*	Triphosphoribosyl-dephospho-CoA synthase [44].

**Table 3:** List of HOCl-resistance genes from the genome-wide screening.

Biological Functions	Genes
Stress responses	*cheW*, *dacB*, *frmB*, *gloA*, *groL*, *hyaE*, *hyaF*, *hybG*, *inaA*, *otsA*, *rfbC*, *sanA*, *sbmC*, *solA*, *ssuD*, *ycbB (ldtD)*, *yghW*, *yhaK*, *yodA (ZinT)*, *yqjA*
Membrane components and transporters	*appC*, *chbB*, *exbD*, *gspK*, *hyaA*, *hyaC*, *hydN*, *marC*, *mpaA*, *nmpC*, *nrfC*, *rfe*, *slyB*, *tap*, *ybhC*, *ybhR*, *ybiM*, *ybiO*, *yceJ*, *ydgK*, *yfdY*, *yggR*, *ygjE (ttdT)*, *yhiP (dtpB)*, *yoaE*, *yoeE*, *ypfJ*, *yrbB*
Metabolism	*acnA*, *agaS*, *aspC*, *bioA*, *cobS*, *cynT*, *dadA*, *hisA*, *ilvA*, *leuA*, *mtlD*, *nagD*, *paaG*, *prpE*, *purB*, *sucC*, *ubiD*, *upp*, *ycdK (rutC)*, *ygbL*
DNA replication, gene expression and regulation	*agar*, *gyrA*, *lhr*, *rnc*, *rpoS*, *rsuA*, *trmU*, *uvrY*, *yccK (tusE)*, *ydaV*, *ydcP (rlhA)*, *ydiP*, *ydjF*, *yhaJ*, *yhdJ*, *ykgM*, *ymfG (xisE)*, *yneJ*
Cell division & mobility	*flip*, *fliS*, *motB*, *ydeQ*, *yedQ (dgcQ)*, *ygcF (queE)*, *yneF (dgcF)*
Other and unknown	*dsrB*, *elaA*, *smf*, *sprT*, *yagM*, *ybbV*, *yccJ*, *ycgY*, *yddJ*, *ydfB*, *ydfD*, *ydhL*, *yeeX*, *yfiH (pgeF)*, *yhcG*, *ykfC*, *ymfE*, *yncH*, *yncM*, *ynfN*, *ytfI*

**Table 4:** List of genes mediating stronger HOCl-resistance.

Gene	Known or Projected Functions
*prpE*	Propionate-CoA ligase; catalyzes the synthesis of propionyl-CoA from propionate and CoA [31].
*frmB*	S-formylglutathione hydrolase; hydrolyzes S-formylglutathione to glutathione and formate [45].
*rutC*	Putative aminoacrylate peracid reductase [46].
*sucC*	Succinate-CoA ligase (ADP-forming) subunit beta; functions in the citric acid cycle [47].
*ycbB*	Periplasmic L,D-transpeptidase; plays a role in the protective remodeling of peptidoglycan during cell envelope stress [48].
*yccJ*	PF13993 family protein.
*hyaC*	Probable Ni/Fe-hydrogenase 1 b-type cytochrome subunit; functions in anchoring hydrogenase to the membrane [49].
*hyaE*	Hydrogenase-1 operon protein [50].
*tap*	Methyl-accepting chemotaxis protein IV [51].
*yoaE*	UPF0053 inner membrane protein; putative transport protein [52].
*motB*	Motility protein B; comprises the stator element of the flagellar motor complex with MotA [53].
*rnc*	Ribonuclease 3 for rRNA processing [54].
*elaA*	Putative N-acetyltransferase.
*yfdY*	DUF2545 domain-containing protein [55].
*exbD*	A component of the energy-transducing Ton system [56].
*ilvA*	Threonine deaminase; carries out the first step in the synthesis of isoleucine [41].
*yhdJ*	Overexpression of YdhJ leads to methylation of genomic DNA at the NsiI recognition sequence (5′-ATGCAT-3′) [57].
*ytfI*	Uncharacterized gene.
*ybhR*	One of two integral membrane subunits of a putative ABC exporter [58].
*yedQ*	A probable inner membrane protein whose expression is dependent on σS under a number of stress conditions [59].
*marC*	An inner membrane protein with six predicted transmembrane domains [52].
*sanA*	Multi-copy expression of sanA complements the vancomycin sensitivity of an *E. coli* K-12 mutant with outer membrane permeability defects [60].
*rsuA*	Pseudo-uridine synthase that is responsible for pseudouridylation of 16S rRNA at position 516 [61].

**Table 5:** List of identified genes mediating both H_2_O_2_ and HOCl-resistance.

Gene	Known or Projected Functions
*leuA*	2-isopropylmalate synthase; involved in the first committed step in leucine biosynthesis [29].
*prpE*	Propionyl-CoA synthetase; catalyzes formation of propionyl-CoA via the methylcitrate cycle [31].
*sucC*	β subunit of succinyl-CoA synthetase [47].
*ybhC*	An outer membrane lipoprotein [34].
*hyaA*	Small subunit of hydrogenase-1; contains a unique proximal [4Fe-3S] cluster that is essential for oxygen tolerance [63].
*ydeQ*	Uncharacterized gene.
*cheW*	Chemotaxis protein; in the ternary receptor complexes of two-component signaling pathways [64].
*motB*	Motility protein B; comprises the stator element of the flagellar motor complex with MotA [53].
*yodA*	Metal-binding protein; may function as a periplasmic zinc chaperone delivering zinc to apo-enzymes in this compartment [65].
*ydfB*	Uncharacterized gene.
*fliS*	Flagellar biosynthesis protein; substrate-specific chaperones of the flagellar export system [66].
*gloA*	Glyoxalase I; catalyzes the first of two sequential steps in the conversion of methylglyoxal to D-lactate [67].
*ydfD*	A lysis protein encoded by the Qin prophage [68].
*slyB*	Outer membrane lipoprotein [69].
*yoeE*	TonB-dependent receptor plug domain-containing protein; may be regulated by Fur regulon [70].
*sbmC*	DNA gyrase inhibitor; protects cell from DNA damage cause by DNA-bound gyrase [71].
*upp*	Uracil phosphoribosyltransferase; a pyrimidine salvage enzyme that catalyzes the synthesis of uridine 5′-monophosphate from uracil and 5-phospho-α-D-ribose 1-diphosphate [72].
*yhaJ*	DNA-binding transcriptional activator; a member of the LysR protein family [73].
*agaR*	DNA-binding transcriptional repressor [74].
*exbD*	A component of the energy-transducing Ton system [56].
*agaS*	Putative galactosamine-6-phosphate deaminase/isomerase.
*ilvA*	Threonine deaminase; carries out the first step in the synthesis of isoleucine [41].
*ubiD*	3-octaprenyl-4-hydroxybenzoate decarboxylase; an enzyme of the ubiquinol-8 biosynthesis pathway that catalyzes the decarboxylation of 3-octaprenyl-4-hydroxybenzoate [75].

## Data Availability

Data is contained within the article and supplementary material.

## Supplementary Materials

The following are available online at https://www.mdpi.com/article/10.3390/antiox10060861/s1, Figure S1: High-resolution image of the protein–protein interaction map for H_2_O_2_ resistance genes; Figure S2: High-resolution image of the protein–protein interaction map for HOCl resistance genes.
