# Supplementary figures and images for: Genome-Wide Screening of Oxidizing Agent Resistance Genes in Escherichia coli
Source: Antioxidants (Basel). 2021 May 27;10(6):861. doi: 10.3390/antiox10060861 (PMC8228696; doi:10.3390/antiox10060861)

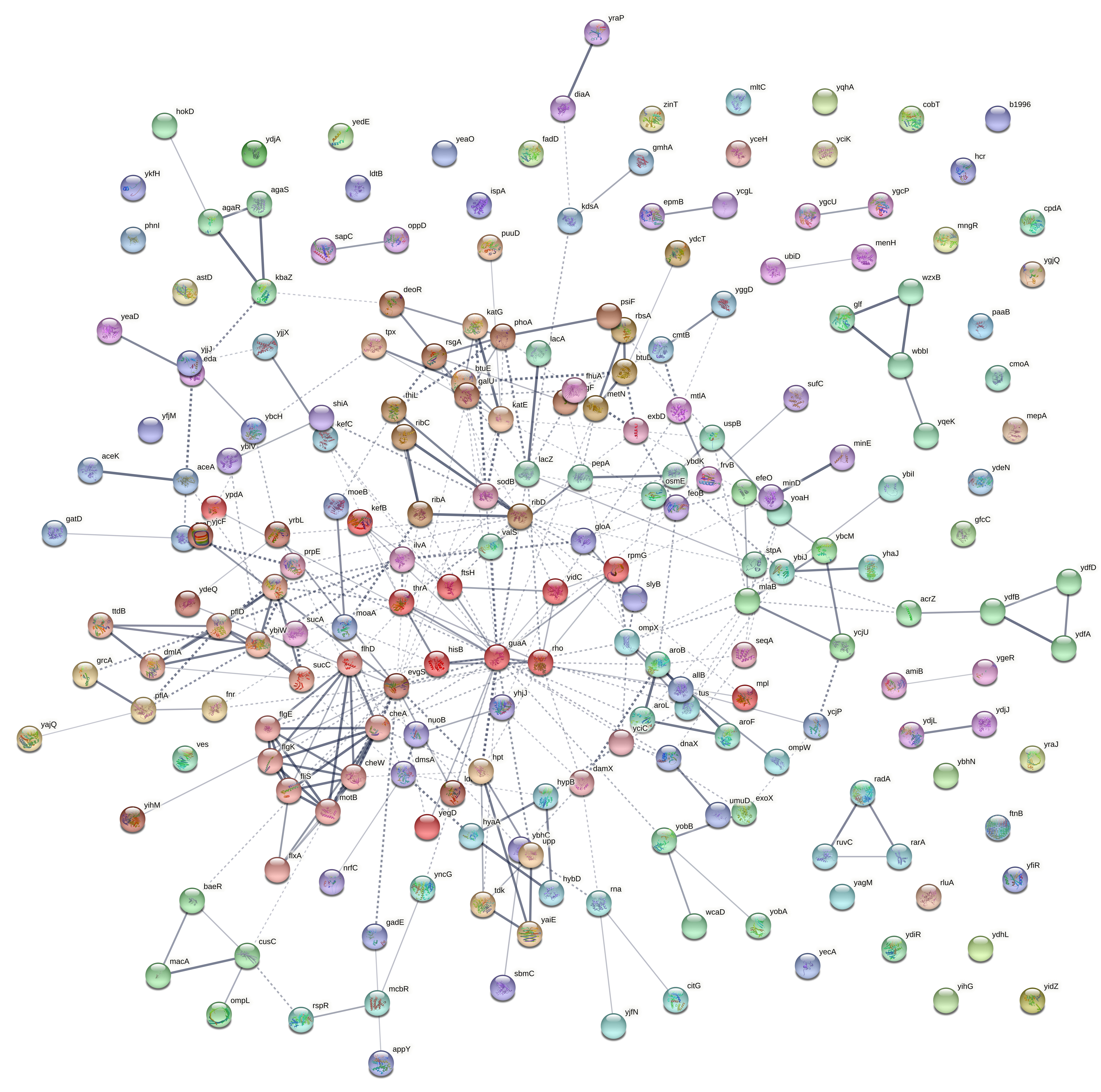

Supplement: Supplementary file 1 [file antioxidants-10-00861-s001.zip › Figure S1. H2O2 string map.png]

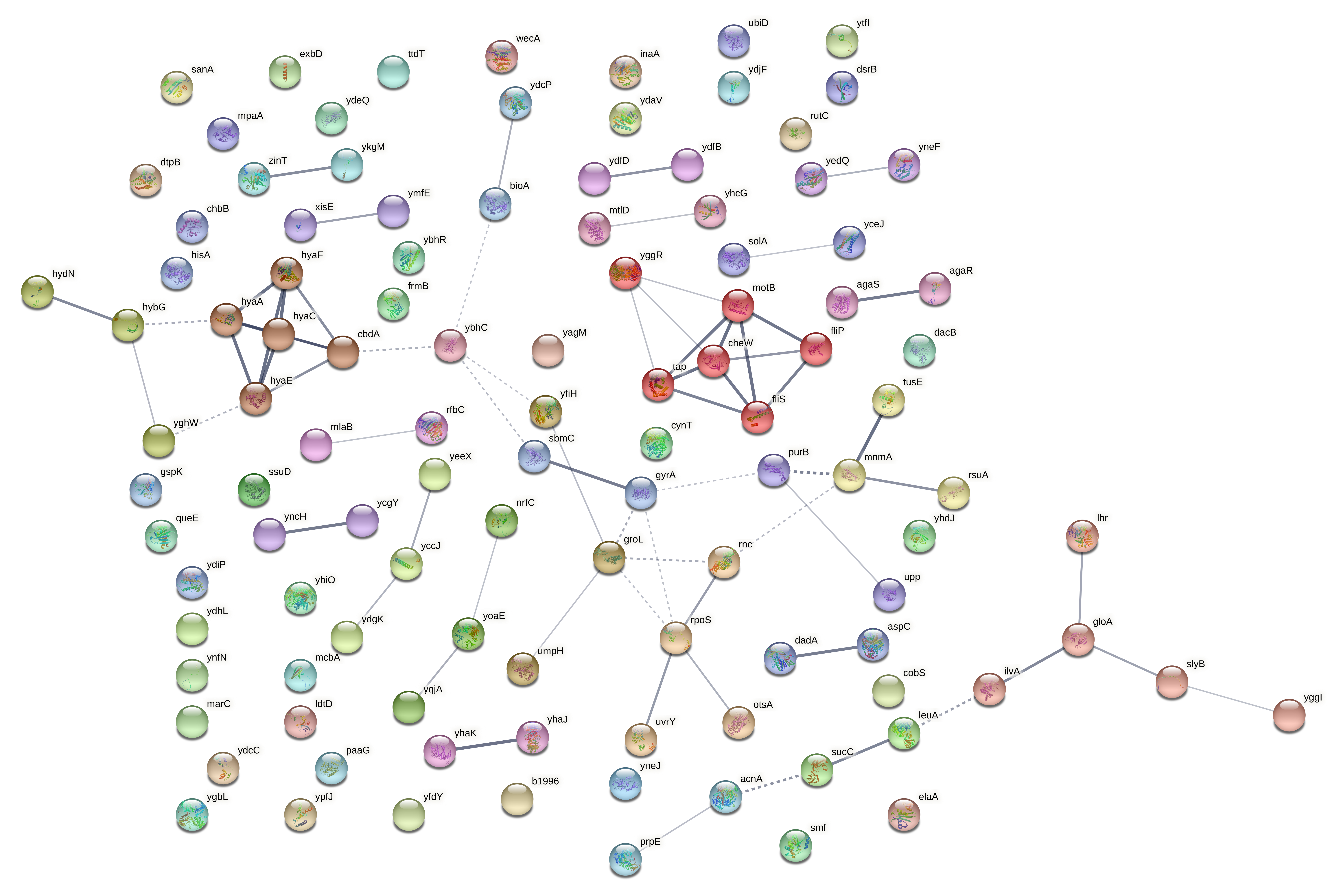

Supplement: Supplementary file 1 [file antioxidants-10-00861-s001.zip › Figure S2. HClO string map.png]
